# Combination therapy with immune checkpoint inhibitors (ICIs); a new frontier

**DOI:** 10.1186/s12935-021-02407-8

**Published:** 2022-01-03

**Authors:** Somayeh Vafaei, Angelina O. Zekiy, Ramadhan Ado Khanamir, Burhan Abdullah Zaman, Arman Ghayourvahdat, Hannaneh Azimizonuzi, Majid Zamani

**Affiliations:** 1 Department of Molecular Medicine, Faculty of Advanced Technologies in Medicine, Iran University of Medical Sciences, Tehran, Iran; 2 Department of Prosthetic Dentistry, I. M. Sechenov First Moscow State Medical University, Moscow, Russia; 3 Internal Medicine and Surgery Department, College of Veterinary Medicine, University of Duhok, Kurdistan Region, Iraq; 4 Basic Sciences Department, College of Pharmacy, University of Duhok, Kurdistan Region, Iraq; 5 Medicine Faculty, Hacettepe University, Ankara, Turkey; 6 Medicine Faculty, Cerrahpasa University, Istanbul, Turkey; 7 Department of Medical Laboratory Sciences, Faculty of Allied Medicine, Infectious Diseases Research Center, Gonabad University of Medical Sciences, Gonabad, Iran

**Keywords:** Immune-checkpoint inhibitors (ICIs), Tumor microenvironment (TME), Resistance, Combination therapy, Immune cells

## Abstract

Recently, immune checkpoint inhibitors (ICIs) therapy has become a promising therapeutic strategy with encouraging therapeutic outcomes due to their durable anti-tumor effects. Though, tumor inherent or acquired resistance to ICIs accompanied with treatment-related toxicities hamper their clinical utility. Overall, about 60–70% of patients (e.g., melanoma and lung cancer) who received ICIs show no objective response to intervention. The resistance to ICIs mainly caused by alterations in the tumor microenvironment (TME), which in turn, supports angiogenesis and also blocks immune cell antitumor activities, facilitating tumor cells' evasion from host immunosurveillance. Thereby, it has been supposed and also validated that combination therapy with ICIs and other therapeutic means, ranging from chemoradiotherapy to targeted therapies as well as cancer vaccines, can capably compromise tumor resistance to immune checkpoint blocked therapy. Herein, we have focused on the therapeutic benefits of ICIs as a groundbreaking approach in the context of tumor immunotherapy and also deliver an overview concerning the therapeutic influences of the addition of ICIs to other modalities to circumvent tumor resistance to ICIs.

## Introduction

**Fig. 1:**
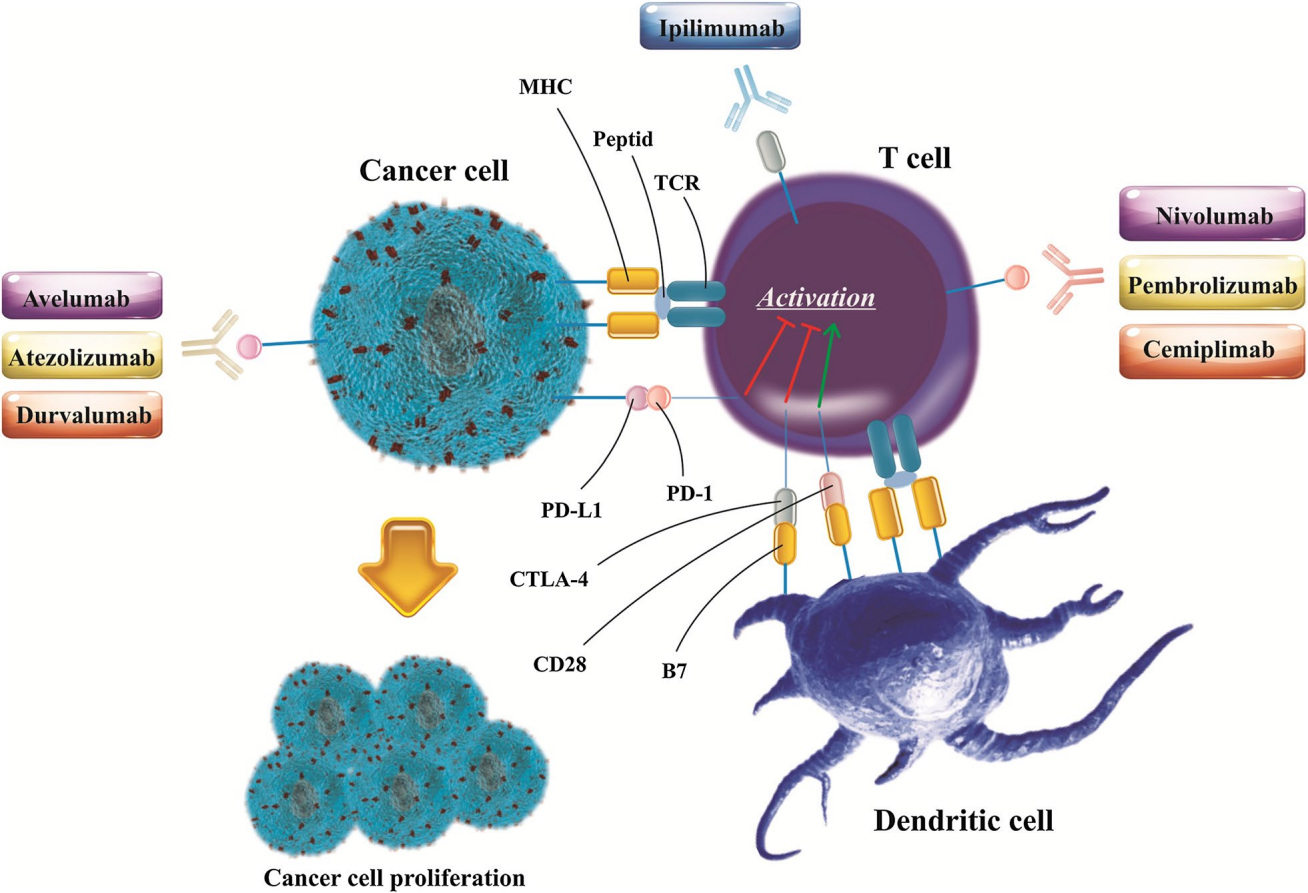
The FDA-approved immune checkpoint inhibitors (ICIs). PD-1 inhibitors nivolumab, pembrolizumab, cemiplimab, PD-L1 inhibitors atezolizumab, avelumab, and durvalumab, and also CTLA-4 inhibitor ipilimumab have been approved as most eminent ICIs to treat a myriad of cancers

During the last two decades, tumor immunotherapy has evolved the clinical management of a diversity of tumors even with undesired prognoses [1, 2]. As one of the most eminent eras in the context of tumor immunotherapy, immune-checkpoint inhibitors (ICIs) have engendered remarkable therapeutic outcomes as a result of their broad bioactivity across numerous histological tumor types along with their durable anti-tumor impacts [3, 4]. Among the checkpoint-blocking strategies, inhibition of the cytotoxic-T-lymphocyte-associated protein 4 (CTLA-4 or CD152) and also blocking the interfaces between programmed cell death 1 (PD-1 or CD279) and programmed cell death ligand 1 (PD-L1 or CD274 or B7 homolog 1) has gained increasing attention [5]. Due to the substantial homology to the costimulatory molecule CD28, CTLA-4 can bind B7 molecules on antigen-presenting cells (APCs) with much higher affinity and also avidity than CD28, averting the activation of T cell responses [6]. The evidence regarding the CTLA-4 activities offered the concept that dampening its activities could enable durable T cell responses [7]. Then, accumulating evidence supported the responding notion, and after than much effort was spent to produce ipilimumab, a monoclonal antibody (mAb) targeting human CTLA-4 [8]. Irrespective of inhibition of the costimulation, CTLA-4 inhibitors can also attenuate regulatory T (Treg) cell recruitment into tumor tissue due to the high expression of CTLA-4 on the surface of Treg [9]. Negative regulation of Tregs population in the tumor microenvironment (TME), in turn, largely improves the infiltration as well as anti-tumor activities of tumor-infiltrating lymphocytes (TILs), in particular, cytotoxic T lymphocytes (CTL) [10]. On the other hand, the PD-1 functions as a critical immune checkpoint were documented upon detecting its central ligand, PD-L1, which is found on multiple cell types such as tumor cells, immune cells, epithelial cells, and endothelial cells [11]. Similar to CTLA-4, the PD-1is expressed on induced T cells and contributes to the down-regulation of signaling complicated in antigen recognition by the T cell receptor (TCR) [12]. PD-L1 expression is in association with exposure to interferon-γ (IFN-γ) for example following anti-tumor T helper type 1 (Th1) cell responses, and could ultimately ease tumor cell’s escape from T cell antitumor immunity [13–15]. Like anti-CTLA-4 antibody ipilimumab, PD-1/PD-L1 inhibitors, surrounding nivolumab, pembrolizumab, cemiplimab, atezolizumab, avelumab, and durvalumab have gained approval from United States Food and Drug Administration (FDA) during the last decade (Fig. 1) [16, 17]. However, tumor resistance to ICIs [18, 19] and also treatment-associated toxicities [20] impede their clinical utility. Recent reports have shown that objective response rate (ORR) in melanoma patients treated with PD-1 inhibitors is only 33%, and also more than 70% of non-small-cell lung carcinoma (NSCLC) patients exhibit no response to ICIs [21]. It has been evidenced that TME in association with other factors supports chronic inflammation, improves immunomodulation, and concomitantly aids pro-angiogenic intratumoral microenvironment, and thereby entices tumor cells evasion from recognition and succeeding elimination by host immunosurveillance [22, 23]. Accordingly, several studies have exhibited that combination therapy with ICIs plus other therapeutic approaches, such as chemotherapy [24–26], radiotherapy [27, 28], cancer vaccines [29–31], anti-angiogenic agents [32–34], HER-2 targeted therapies [35] and also CXCR4 blockade therapy [36, 37] can efficiently circumvent tumor resistance to ICI therapy.

In the present review, we deliver an overview about the therapeutic merits of ICIs as a pioneering tactic in tumor immunotherapy and also discuss recent reports evaluating the combined use of ICIs with other conventional approaches to overcome tumor resistance to ICI, with a particular concentration on last decade in vivo reports.

## The rationality of ICIs therapy

Communication between immune checkpoints and their responding ligands abrogates T cell activation and resultant anti-tumor immunity by targeting a myriad of signaling axes, in particular, phosphatidylinositol-3-kinase (PI3K)/Akt pathway [38]. As a result, NF-kB and mTOR activation and also IL-2 and Bcl-xL expression are negatively affected in activated T cells [39]. Such events eventually hinder physiological immune reactions against tumor-associated antigens (TAAs). Notably, immune checkpoints and the related ligands are mainly upregulated in the TME and also on the surface of tumor cells, and so underlies blockade of anti-tumor immune response [40, 41].

As known, CD80 (B7-1) and CD86 (B7-2) co-stimulation by CD28 delivers vital stimulatory signals, which eases T cell proliferation and differentiation throughout the induction phase of immunological response [42]. The CTLA-4 co-inhibitory receptor is largely demonstrated on lately activated T cells and creates interfaces with the same ligands as CD28 but with higher affinity [43, 44]. Interrelation between CTLA-4 and CD80/86 impedes T cell activation by both suppressing the formation of a communication between CD80/CD86 and CD28, and also transmitting suppressive signals [45, 46]. Structurally, CTLA-4 includes a unique YVKM motif at the cytoplasmic domain, which brings about inhibitory signaling upon interaction with the Src homology 2 domain-containing protein tyrosine phosphatase 2 (SHP-2) [7]. CTLA-4 inhibits T-cell responses by cell-intrinsic and extrinsic pathways. Intrinsic events involve the suppression of protein translation and cytokine receptor signaling through the induction of the recruitment of phosphatases and ubiquitin ligases [47]. Besides, cell-extrinsic actions comprise the competition for CD28 in binding to CD80/86, the removing CD80/86, secretion of suppressive indoleamine (2,3)-dioxygenase (IDO), and also targeting Treg activities [47]. Other in vivo reports deliver the proof of the hypothesis that CTLA-4 can adjust T-cell infiltration into allografts as well as tumors [48]. Unsurprisingly, elevated levels of CTLA-4 in association with poor prognosis has been found in NSCLC [49–51], breast cancer [52, 53], nasopharyngeal carcinoma [54], small cell lung cancer (SCLC) [55], prostate cancer [56], thymoma [57], melanoma [58, 59], colorectal cancer (CRC) [60], glioblastoma [61] and osteosarcoma [62].

Anti-tumor T cells following acquirement of cytokine-producing and cytolytic effector competencies can undergo additional negative regulation by an interaction between PD-1 on such cells with PD-L1 on tumor cells or tumor-associated antigen-presenting cell (APC) in the TME [63, 64]. Interaction between PD-L1expressing tumor cells or APC and PD-1 expressing T cells leads ultimately to eliciting signaling by cytoplasmic tail of PD-1, facilitating T cell exhaustion. The cytoplasmic tail of PD-1 includes two tyrosine-based structural motifs, an immunoreceptor tyrosine-based inhibitory motif (ITIM) (V/L/I/XpYXX/L/V) and an immunoreceptor tyrosine-based switch motif (ITSM) (TXpYXXV/I) [65]. The PD-1 suppressive activities depend on the ITSM phosphotyrosine, which in turn, potentiates the recruiting SHP-2 and suppressing downstream signaling pathways like CTL-4 [65, 66]. Various tumors apply this mechanism by up-regulation of PD-L1 which often relates to unfavorable prognosis. Further, expression of PD-1 on some tumor cells has also recently been elucidated [67]. Indeed, interfaces between PD-L1 on tumor cells with PD-1 on immune cells sustain immune escape and tumor development more chiefly by suppression of cytotoxic T lymphocyte (CTL) effector function [68]. Improved expression of PD-L1 on tumors has been validated to intensely correlate with advanced disease state and unfavorable prognosis in melanoma, breast, gastric, ovarian, liver, kidney, pancreatic, and also bladder cancer [68].

Given that ICIs with the goal of targeting CTLA-4, PD-1, or PD-L1 can dampen immune checkpoints-induced inhibitory impacts on T cells biological processes, making further progress to evolve novel ICIs for broader types of malignancies is urgently justified.

## FDA-approved ICIs

### CTLA-4 inhibitors

The monoclonal antibody ipilimumab which targets CTLA-4 has been approved on March 25, 2011, to treat patients with metastatic melanoma [8]. It is also used in combination with nivolumab for the treatment of advanced renal cell carcinoma (RCC) [69], microsatellite instability-high (MSI-H) or mismatch repair deficient (dMMR) metastatic CRC [70], hepatocellular carcinoma (HCC) [71], NSCLC [72], and malignant pleural mesothelioma (MPM) [73]. The most common adverse events correlated with ipilimumab are immune related adverse events (irAEs), and both anti-cancer and irAE reactions. During the last decade, some clinical trials have indicated that monotherapy with ipilimumab (10 mg/kg) in patients with advanced melanoma could result in improved OS rate [74], and a durable objective response [75]. In addition to monotherapy with ipilimumab, this CTLA-4 inhibitor combined nivolumab led to longer progression-free survival (PFS) and a higher objective response rate (ORR) in a phase 3 trial in patients with advanced melanoma (NCT01844505) [76]. Importantly, the OS rate at 3 years was 58% in the nivolumab plus ipilimumab group and 52% in the nivolumab group, while was 34% in the ipilimumab group [76]. Besides, irAEs happened in 59% of the patients in the nivolumab plus ipilimumab group, in 21% of patients in the nivolumab group, and 28% of patients in the ipilimumab group [76]. Thereby, combination therapy showed superiority over monotherapy with ipilimumab or nivolumab in terms of efficacy, while a higher rate of the occurrences of irAEs dampens its clinical use [76]. Further, the combination of nivolumab and ipilimumab induced a deep enhancement in proliferation and activation of T cells in MPM patients (NCT03048474) [77]. Patients that responded to treatment with nivolumab plus ipilimumab had low densities of naive CD8 T cells and conversely high densities of effector memory CD8 T cells and granzyme-B and interferon-γ producing T cells [77]. Another trial on 108 patients also revealed that monotherapy with nivolumab and also combination therapy with nivolumab plus ipilimumab demonstrated promising anti-tumor activities in relapsed patients with MPM, without unexpected toxicity [73]. Meanwhile, 44% of patients in the nivolumab group and 50% of patients in the nivolumab plus ipilimumab group experienced 12-week disease control [73]. As well, a phase 3 trial on patients with advanced NSCLC verified the superiority of combination therapy with nivolumab plus ipilimumab on chemotherapy, as shown by higher median OS rate in combination therapy group than chemotherapy group (17.1 months versus 14.9 months. Meanwhile, the median duration of response (DOR) was 23.2 months with ipilimumab plus nivolumab and 6.2 months with chemotherapy (NCT02477826) [72]. These findings provided clear evidence implying that combination therapy with ipilimumab plus nivolumab has superiority over chemotherapies due to the lower safety concerns and higher activities in NSCLC [72]. The safety and efficacy of combination therapy with nivolumab plus ipilimumab also was indicated in dMMR/MSI-H metastatic CRC [70]. Accordingly, PFS rates were 76% (9 months) and 71% (12 months) and respective OS rates were 87% and 85% [70]. Correspondingly, amelioration was observed in patients, such as functioning, symptoms, and quality of life, and also intervention showed manageable ir-AEs [70, 78].

### PD-1 inhibitors

Nivolumab, pembrolizumab, and cemiplimab, well-known PD-inhibitors, are fully human IgG4 mAb and have demonstrated capable potential to treat advanced melanoma and NSCLC patients [79]. Apart from combination therapy with ipilimumab, monotherapy with nivolumab also is indicated for gastric cancer, and classic Hodgkin's lymphoma (cHL) therapy [80]. Pembrolizumab has been used for the treatment of patients with metastatic melanoma and NSCLC [81], metastatic bladder cancer [82], head and neck squamous cell carcinomas (HNSCC) [83], refractory cHL [84], and metastatic ESCC [85]. Further, cemiplimab has been approved for metastatic cutaneous squamous cell carcinoma (CSCC) therapy [86].

Recently, study of the efficacy and safety of nivolumab in 440 patients with wild-type BRAF and mutant BRAF metastatic melanoma showed that nivolumab administration caused improved ORR regardless of the PD-L1 status of the tumor [87]. As well, the durable response rate (DRR) was 14.8 months for wild-type BRAF and 11.2 months for mutant BRAF. Accordingly, it was speculated that nivolumab has comparable efficacy and safety consequences in patients with wild-type or mutant BRAF [87]. Likewise, pembrolizumab showed great potential for the treatment of advanced melanoma regardless of BRAF V600E/K mutation status [88]. Besides, a meta-analysis investigating the efficacy and safety of nivolumab for advanced NSCLC patients evidenced the strong capacity of administration of nivolumab (3 mg/kg), as demonstrated with ameliorated ORR, OS, and also PFS [89]. Moreover, patients with positive PD-L1 expression showed a more favorable response to nivolumab [89]. Moreover, nivolumab could also elicit long-term clinical merits and a favorable tolerability profile than docetaxel, a taxoid antineoplastic agent, in patients with advanced NSCLC [90]. Meanwhile, OS rates with nivolumab versus docetaxel were 23% versus 8% in squamous NSCLC and 29% versus 16% in nonsquamous NSCLC [90]. Also, combination therapy with pembrolizumab and radiotherapy (RT) could support improved PFS and OS compared with monotherapy with RT with an acceptable safety profile in NSCLC patients [91]. The median OS was 10.7 months versus 5.3 months in the pembrolizumab plus RT group versus RT alone. Thereby, it was simplified that application of ICIs along with RT may be considered as an effective strategy in patients with NSCLC or even other tumors [91]. Pembrolizumab could also improve OS in patients with locally advanced or metastatic urothelial carcinoma (UC), according to Sundahl et al. reports [92]. As well, results from another trial on 370 UC patients revealed that pembrolizumab inspires acceptable DRR in cisplatin-ineligible patients (NCT02335424) [93]. This monoclonal antibody also elicited significant antitumor activity, as evidenced by improved ORR, with manageable toxicity in HNSCC [94]. Notwithstanding, administration of pembrolizumab was not able to affect OS and PFS in HNSCC patients compared with standard of care (SOC) chemotherapy regimens (cetuximab, docetaxel, or methotrexate) [94]. Moreover, pembrolizumab alone or in combination with platinum and 5-FU could be considered as first-line standards of care for HNSCC (NCT02358031) [95]. Besides, Chen and his colleagues showed that pembrolizumab could affect the ORR as well as complete response rate (CRR) in patients with cHL (NCT02453594) [96]. On the other hand, cemiplimab has shown substantial antitumor functions with a manageable safety profile in patients with metastatic CSCC [97, 98]. The most common adverse events regardless of attribution during or after treatment of CSCC patients with cemiplimab (3 mg/kg) are fatigue (27.0%) and diarrhea (23.5%) [99]. As well, it was suggested that cemiplimab was correlated with benefits in OS and PFS in CSCC patients versus EGFR inhibitors and pembrolizumab, signifying its great potential in treating CSCC patients [100]. Cemiplimab monotherapy also could bring about higher OS and PFS than chemotherapy with platinum-based compounds in patients with advanced NSCLC, and so suggesting a potential new therapeutic approach for this patient population [101].

### PD-L1 inhibitors

Three anti-PD-L1 antibodies have gained approval from the FDA: atezolizumab (IgG4 mAb), and also durvalumab and avelumab, which are IgG1 mAb [102]. Since 2016, atezolizumab as the first FDA-approved PD-LI inhibitor has been approved for advanced or metastatic UC patients [103]. Also, it has been indicated for metastatic NSCLC patients whose malignancy progressed throughout or upon platinum-based compound therapy [104]. Moreover, atezolizumab plus angiogenesis inhibitor bevacizumab is used for metastatic HCC patients therapy [105], and also in combination with mitogen-activated extracellular kinase (MEK) inhibitor cobimetinib and B-Raf enzyme inhibitor vemurafenib is applied for the treatment of patients with metastatic melanoma [106]. Since 2017, durvalumab has been approved for the treatment of advanced or metastatic UC [107] as well as metastatic Merkel cell carcinoma (MCC)[108]. Durvalumab plus etoposide and either carboplatin or cisplatin are now used as a first-line treatment for advanced NSCLC therapy [109]. Since 2017, avelumab has been utilized for MCC [108] and metastatic UC therapy [110]. Moreover, since 2019, avelumab plus tyrosine kinase inhibitor axitinib is used as the first-line treatment of patients with advanced RCC [111].

Currently, a phase 3 trial indicated that atezolizumab could stimulate objective responses in metastatic UC with or without platinum-based chemotherapy, as shown by improved PFS and also acceptable safety profile [112]. Also, atezolizumab in patients with previously treated advanced NSCLC exhibited significant amelioration in OS versus docetaxel (13.3 versus 9.8 months) without unexpected toxicities [113]. As well, this ICI plus carboplatin and paclitaxel showed superiority over chemotherapy alone in terms of improved OS and PFS in advanced NSCLC patients according to West et al. reports [114]. Gutzmer et al. also found that combination therapy with atezolizumab plus targeted therapy with vemurafenib and cobimetinib was safe and tolerable and considerably promoted PFS in patients with BRAFV600 mutation-positive advanced melanoma [106]. Nonetheless, some adverse events such as increased blood creatinine phosphokinase, lipase and alanine aminotransferase, diarrhea, rash, arthralgia, pyrexia were shown [106]. Another PD-L1 inhibitor, durvalumab, has demonstrated clinical benefit in patients with locally advanced or metastatic UC. Durvalumab administration (10 mg/kg) could provoke improved ORR, OS, and PFS concomitant with the excellent safety profile in patients with UC (NCT01693562) [115]. Besides, durvalumab plus platinum-etoposide supported improved OS versus platinum-etoposide therapy (13.0 months versus 10.3 months) in patients with SCLC without any significant difference respecting grade 3 or 4 adverse events percentages between two groups [109]. As well, this PD-L1 inhibitor resulted in improved OS and PFS along with DOR more obviously patients with PD-L1 expressing tumors [116]. Apart from efficacy, another trial evaluating long-term safety supported that avelumab administration had no new or unexpected adverse events and no treatment-related deaths in MCC patients during 3 years follow-up [117]. This evidence reflects the capacities of avelumab as a SOC treatment option for MCC [117]. Avelumab also in combination with axitinib is now described as first-line treatment for patients with advanced RCC with manageable safety profile and substantial tolerability [111, 118]. In advanced RCC, addition of the avelumab to axitinib also improved PFS compared with sunitinib, an FDA-approved VEGFR inhibitor for RCC patients [119].

## Corresponding mechanism complicated in tumor resistance to ICIs

It is now generally documented that tumor cells make close interfaces with the ECM, stromal cells, and also immune cells which typically exist in TME. Such cells in TME support evolving chronic inflammation, enhancing immunomodulation, and simultaneously providing a pro-angiogenic intratumoral microenvironment, and thus ease tumor cells escape from recognition and subsequent removal by host immunosurveillance [120, 121]. For eradication of malignant cells, T cells are required to be efficiently induced by dendritic cells (DCs) in peripheral lymph nodes, home to the malignant tissue, extravasate from malignant tissue blood vessels, and finally infiltrate barricades (such as stromal tissue) to encounter cancer cells [122, 123]. Developing tumors mainly barricade these necessities for T cell immunosurveillance for preventing immune cell-elicited tumor eradication. Given that the efficacy of ICIs treatment is principally inspired by T cells, such competent immune escape may ultimately bring about failures in ICIs therapy. A promotion in PD-L1 in the TME by malignant cells and also APCs is thought to be the most communal approach by which malignant cells bypass immune surveillance [124, 125]. The tryptophan catabolism inside the TME also is contributed to the negative regulation of anti-tumor immune responses. In TME, tryptophan catabolism induced by the IDO, which is largely expressed by myeloid-derived suppressor cells (MDSC) and tumor cells, results in making some immunosuppressive metabolites (e.g., kynurenine) [126]. Bothe kynurenine functions and also exhaustion of the vital amino acid tryptophan impede T cell’s clonal expansion and may entice either T cell anergy or apoptosis [126]. Owing to this fact, the combined effects of IDO inhibitors and ICIs have been speculated as a rational plan to provoke TILs and their functional aptitudes in the TME. This intervention can facilitate removing both IDO-expressing and IDO-nonexpressing poorly immunogenic malignant cells [127]. Likewise, the existence of regulatory T cells (Treg cells), T helper 2 (TH2) cells, and MDSCs in TME is an additional impediment, compromising the efficacy of ICIs therapies by suppressing CTL- and T helper 1 (TH1) cell-mediated tumor immunosurveillance [128, 129]. Exhaustion of such cell types has experimentally been exposed to augment anti-tumor immune responses defeating resistance to ICI [21]. Besides, an intrinsic mechanism such as up-regulation of the tumor-inducing WNT-β-catenin signaling pathways may avert TILs and CD103 + DC infiltration into the TME. As evidenced in melanoma, it appears that β-catenin activation could suppress the expression of chemokine chemokine (C–C motif) ligands 4 (CCL4), which is mainly complicated in immune cell infiltration into TME [130, 131]. Besides, loss of phosphatase and tensin homolog (PTEN) is allied with improved levels of CCL2 and vascular endothelial growth factor (VEGF), reduced infiltration of T cells, and finally resistance to PD-1 inhibitors [132]. Thereby, stimulating DCs migration, maturation, and activation by blockade of immunosuppressive factors, such as VEGF, IL-10, and TGF-β efficiently enables sufficient T-cell priming and cooperation with ICI. As well, cyclooxygenase (COX) expression by tumor cells can hinder tumor cell immunosurveillance as a result of up reregulation of the prostaglandin E2 (PGE2) expression, preparing an inflammatory environment for tumor growth [133, 134]. Moreover, COX-2 overexpression mainly improves Treg trafficking into TME. The metabolic interaction between the transformed cells and immune cells also may give rise to the poor response to treatment with ICI, as evidenced by the study of the tumor and immune cell glucose and glutamine metabolism [135]. In fact, glucose and glutamine metabolism up-regulate the PD-L1 expression in transformed cells by the positive regulation of epidermal growth factor receptor (EGFR)/ extracellular signal-regulated kinase (ERK)/C-Jun pathway [135]. Hence, inhibiting tumor glucose or glutamine metabolism by therapeutic molecules in combination with PD-1/PD-L1 blockade therapies may defeat tumor cell resistance to ICIs. On the other hand, janus kinase (JAK) 1/2 loss-of-function mutations are other tools exploited by tumor cells to trigger primary resistance to PD-1 inhibitors by down-regulation of PD-1 expression [136].

## Combination therapy using ICIs

The FDA approved atezolizumab and durvalumab for use in combination with chemotherapy for first-line treatment of patients with advanced SCLC. These approvals were rendering consequences derived from two randomized controlled trials, IMpower133 (atezolizumab) [137] and CASPIAN (durvalumab) [109]. These trials revealed increases in OS with anti-PD-L1 antibodies when used in combination with platinum-based chemotherapy as compared with chemotherapy alone [138]. Atezolizumab has also been approved as a first-line NSCLC irrespective of PD-L1 expression in combination with chemotherapy and bevacizumab [139].

### ICIs with chemotherapy

Recent studies have shown that the combined use of cyclophosphamide, ICI, and vinorelbine could stimulate APC recruitment and also activation, and so hurdle local and metastatic TNBC growth mainly by T-cell-mediated influences in vivo [140]. The intervention, in fact, resulted in activating APCs, increasing intratumoral CD8 + T cells, and also promotion of the progenitor exhausted CD8 + T cells [140]. Also, anti-PD-1 and anti-PD-L1 inhibitors showed synergistic anti-tumor effects with vinorelbine, cyclophosphamide, and fluorouracil (5-FU) in vivo [141]. A study in mice models of breast cancer (BC) and B-cell lymphoma (BCL) revealed that cyclophosphamide heightened circulating MDSC, whereas vinorelbine, cyclophosphamide, and also 5-FU diminished circulating APCs [141]. Vinorelbine and cyclophosphamide, but not 5-FU, also decreased circulating Tregs. However, it was found these events were in association with the administrated dosage of chemotherapeutic agents. For instance, cyclophosphamide (at low doses) and 5-FU (at medium doses) marginally improved circulating Tregs. Further, vinorelbine abridged circulating NKs, whereas low doses of cyclophosphamide and 5-FU improved circulating NKs. These results evidenced the preclinical synergy between chemotherapeutics and anti-PD-L1 [141]. Moreover, monotherapy with CTLA-4 inhibitor and also combination therapy with CTLA-4 inhibitor and either cyclophosphamide or gemcitabine proved their therapeutic effect in BC and also CRC mice model [142]. Notwithstanding, some tumor-bearing mice advanced spontaneous metastases under continuous treatment with combined regimen [142]. Moreover, a phase 1 clinical trial in 15 patients with refractory and metastatic HNSCC indicated that combination therapy with PD-1 inhibitor cemiplimab plus cyclophosphamide, radiation therapy (RT), and granulocyte–macrophage colony-stimulating factor (GM-CSF) could demonstrate acceptable safety profile [143]. However, the regimen resulted in no significant effects compared to the monotherapy with cemiplimab.

Besides, gemcitabine combined with anti- PD-L1 antibody inhibited tumor growth in advanced pancreatic ductal adenocarcinoma (PDAC) murine models [144]. The combined application of PD-L1 inhibitor and gemcitabine improved median OS of treated mice compared to the monotherapy with ICI. Moreover, combination therapy brought about reduced circulating splenic and intratumoral MDSCs, and also M2 macrophages. In contrast, tumor samples from mice administrated with ICI plus gemcitabine had augmented numbers of infiltrating cytotoxic T-cells [144]. Furthermore, addition of the PD-L1 inhibitor to gemcitabine elicited an antitumor response in SCLC mice models by a reduction in M2 macrophage and MDSCs concurrently an enhancement in the expression of the type I interferon beta 1 gene (IFNβ), and CCL5 and CXCL10, largely contributing to the induction of TILs recruitment into tumor tissues [145, 146]. On the other hand, the combination of gemcitabine and PD-1 inhibitors reduced tumor growth and also improved OS in mesothelioma murine model [147]. Combination therapy also improved ORR in two patients with mesothelioma, who were resistant to gemcitabine or PD-1 inhibitor as monotherapy [147]. Likewise, evaluation of the safety and tolerability of the nivolumab as monotherapy or plus gemcitabine and cisplatin as combination therapy in Japanese patients with biliary tract cancer (BTC) was conducted during an open-label, phase 1 clinical trial [148]. Meanwhile, combination therapy exhibited superiority over monotherapy in terms of the improved OS (15.4 versus 5·2 months), enhanced median PFS (4·2 versus 1·4 months), and also achieved ORR (11 patients versus 1 patient) [148]. Likewise, combined use of nivolumab plus gemcitabine and cisplatin induced favorable effects in BTC patients concomitant with some grade 3 or higher adverse events such as thrombocytopenia (56%) and neutropenia (22%) [149]. Importnatly, analysis showed that fitness might be a biomarker for predicting clinical response and also Fas ligand (FasL), monocyte chemoattractant protein-1 (MCP-1/CCL2), and IFN-γ serum levels were associated with prognosis [149]. Other reports also have shown that oxaliplatin as another chemotherapeutic agent could induce robust immunogenic cell death (ICD) in Lewis lung carcinoma (LLC) cells and simultaneously improve DCs and also CTL in LLC tumor tissues, leading to the tumor regression in vivo [150]. Also, combined use of oxaliplatin and PD-L1 inhibitor showed a higher anti-tumor response than monotherapy with oxaliplatin in murine lung carcinoma [150]. It seems that promoted numbers of CTLs in tumor tissue, as evidenced in previous study, rely on the improved expression of T cell-attracting chemokines (CXCL9, CXCL10, and CCL5) as shown in colon cancer MC38 cell bearing mice upon oxaliplatin treatment [151]. Moreover, cisplatin treatment may synergize with PD-1/PD-L1 inhibitors to ameliorate the clinical response, which is principally caused by improved PD-L1 expression [152].

Recently, Chen and coworkers suggested that doxorubicin and cisplatin might stimulate a more valued TME and boost the likelihood of response to anti-PD-1 antibody in TNBC [153]. Furthermore, metronomic paclitaxel could enhance the therapeutic merits of PD-1 in TNBC by altering the tumor immune microenvironment, offering robust proof for the application of this intervention in TNBC patients [154]. Also, paclitaxel improved the efficacy of PD-L1 blockade therapy in tumor animal models and demonstrated a synergistic impact on tumor eradication, metastasis suppression, and also recurrence prevention [155]. Such events might arise from reduced recruitment of Treg cells into TME induced by paclitaxel [155]. In another study, low dose of nanomicelle-encapsulated paclitaxel (nano- paclitaxel) treatment stimulated tumor regression by improving the infiltration and activation of TILs and DCs within tumors [156]. Co-administration of a low dose of nano- paclitaxel and PD-1 inhibitor also provoked CD8 + T cell-dependent antitumor immunity and markedly enhanced the therapeutic efficacy in murine colon cancer CT26 cells and MC38 cell bearing mice [156]. As well, the synergistic effects of PD-1 inhibitor and nanoparticle albumin-bound (nab)-paclitaxel have been recently validated in Chinese patients with refractory melanoma [157]. Accordingly, Li et al. indicated that combination therapy gave rise to the improved ORR and PFS than the control group. Although most patients exhibited adverse events, only 17.2% of participants experienced grade 3 severe adverse events, such as neutropenia (18.8%) [157]. In addition, PD-1/PD-L1 inhibitor plus nab-paclitaxel supported meaningfully longer OS and higher response than ICI monotherapy in patients suffering from the metastatic NSCLC [158]. As well, another clinical trial evidenced the safety and efficacy of doxorubicin chemotherapy plus pembrolizumab in 23 patients with soft tissue sarcomas (STS) [159]. The regimen induced objective response significantly and also prolonged PFS more evidently than monotherapy with pembrolizumab [159].

A summary of conducted studies respecting combination therapy with ICIs and chemotherapy have been listed in (Tables 1 and 4).

**Table 1:** ICI combination therapy with chemotherapy in preclinical models

Tumor	Target IC	Agent (s)	Result (s)	References
Triple-negative breast cancer	PD-1	Cyclophosphamide	Induction of the synergistic effect with ICI through induction of the antigen-presenting cells along with promoting intratumoral CD8 + T cells	[140]
B-cell lymphoma Breast cancer	PD-1 PD-L1	Vinorelbine Cyclophosphamide Fluorouracil	Induction of the synergistic effect	[141]
Breast cancer	CTLA-4	Gemcitabine Cyclophosphamide	Stimulation of tumor regression, while some cases showed the development of spontaneous metastases	[142]
Colon cancer Bladder cancer	PD-1 PD-L1	Methotrexate Vinblastine Doxorubicin Cis-platin Cyclophosphamide	Substantial robust anti-tumor response in vivo	[248]
Gastrointestinal cancer	PD-L1	Gemcitabine	Tumor growth inhibition, reducing MDSCs and M2 macrophages, and improved OS	[144]
Pancreatic ductal adenocarcinoma	PD-1	Gemcitabine	Inspiring the infiltration of Th1 lymphocytes and M1 macrophages along with extended OS	[249]
Small-cell lung carcinoma	PD-1 PD-L1	Gemcitabine	Improving the antitumorigenic CD8 + cytotoxic T cells, DCs, and M1 macrophage populations concurrently decrease in M2 macrophage and MDSCs, and finally enhancement in the expression of the type I interferon beta 1 gene, IFNβ, and chemokines, CCL5 and CXCL10	[145]
Lewis lung carcinoma	PD-1	Gemcitabine	Robust anti-tumor impacts along with suppression of recurrence of LLC by rises in CD8 + and CD4 + T cells proportion	[146]
Mesothelioma	PD-1	Gemcitabine	Tumor regression and improved OS rate	[147]
Lewis lung carcinoma	PD-1	Oxaliplatin	Tumor regression by activation of APCs and TILs	[150]
Colon cancer	PD-1 PD-L1	Cisplatin Oxaliplatin	Promotion of the expression of T cell-attracting chemokines (CXCL9, CXCL10, and CCL5), and Provoking T cell activation and recruitment into TME	[151]
Triple-negative breast cancer	PD-1	Paclitaxel	Instigation of a synergistic effect with ICI through transforming the tumor immune microenvironment	[154]
Triple-negative breast cancer	PD-L1	Paclitaxel	Stimulating tumor regression, metastasis inhibition, and recurrence preventive	[155]
Colon cancer Cervical cancer Lung cancer Melanoma	PD-L1	Paclitaxel	Enhancing the infiltration and function of T cells and DCs within tumors	[156]
Colon cancer Bladder cancer	PD-1 PD-L1	Doxorubicin	Showing the anti-tumor impact of the combination of immunotherapy in the MC38 colon and MB49 bladder models, a lack of response in the 4T1 breast model, and suppression of ICIs potential in the MBT-2 bladder model	[248]
B cell lymphoma	PD-1	Doxorubicin	Verification of the therapeutic capacity of doxorubicin-loaded microbubbles (RDMs) with ICI	[250]
Ovarian cancer	PD-L1	Cisplatin	Prolonged OS of treated mice	[251]
Lung cancer	PD-L1	Cisplatin	Reducing tumor growth	[152]
B cell lymphoma	PD-1	Doxorubicin	Showing synergistic effects with ICI by up-regulation of IFN-γ	[252]
Fibrosarcoma	PD-1	Methotrexate	Notable anti-tumor effect in vivo	[253]

*ICI* immune checkpoint inhibitor, *PD-1* programmed cell death protein 1, *PD-L1* programmed death-ligand 1, *CTLA-4* cytotoxic-T-lymphocyte-associated protein 4, *IFN* interferon, *Tregs* regulatory T cells, *TME* tumor microenvironment, *TILs* tumor-infiltrating lymphocytes, *APC* antigen-presenting cell, *MDSC* myeloid-derived suppressor cells, *OS* overall survival

### ICIs with HER2-targeted therapies

**Fig. 2:**
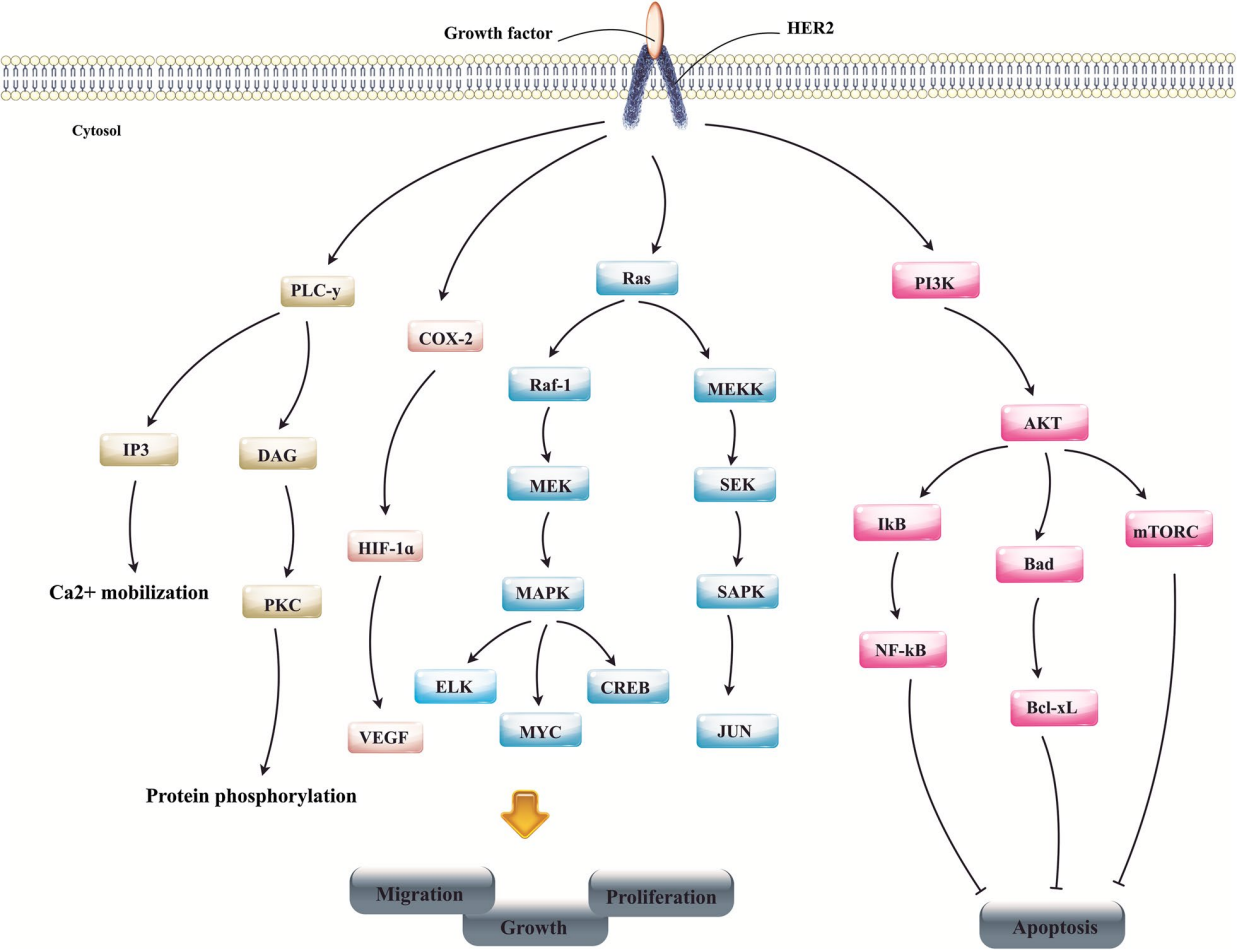
Human epidermal growth factor receptor 2 (HER2) signaling pathway. HER2 and other EGFR family members as RTK located on the cell membrane can responds to multiple ligands, which in turn, result in suppression of tumor cell apoptosis and conversely stimulation of tumor cells migration, proliferation and growth

Human epidermal growth factor receptor (HER) 2 amplification befalls numerous tumor types counting breast, gastric, salivary, vaginal, bladder, CRC endometrial, and cervical. HER2 activation results in the activation of a myriad of oncogenic signaling axes (e.g., PI3K/AKT and Ras/Raf/ERK), thereby improving malignant cell survival, proliferation, migration, and also resistance to immunotherapy (Fig. 2) [160]. Thereby, it is determined as an emerging therapeutic target for breast cancer, and so diversity of ingredients comprising trastuzumab, pertuzumab, lapatinib, neratinib, and trastuzumab emtansine (T-DM1) have been gained approval from the FDA for the treatment of HER2-expressing breast cancer [35]. Notably, HER2-targeted therapy likewise was shown to ameliorate outcomes in HER2-expressing gastric cancer [35].

Recent reports have shown that trastuzumab deruxtecan (DS-8201a), a HER2-targeting antibody, could promote antitumor immunity by enhanced expression of DCs markers, boosted expression of MHC class I in tumor cells, and also the rejection of rechallenged murine HER2-expressing breast cancer cells by adaptive immune cells [161]. Besides, DS-8201a showed a synergistic effect with an anti-PD-1 antibody likely supported by enhanced T-cell mediated anti-tumor activities and upregulated PD-L1 expression [161]. Likewise, combination therapy with DS-8201a and anti-CTLA-4 antibody persuaded more prominent antitumor effects compared with monotherapy with each agent in murine HER2-expressing breast cancer cells mainly by enhanced tumor-infiltrating CD4 + and CD8 + T cells in vivo [162]. Co-administration of T-DM1 with anti-CTLA-4/PD-1 also attenuated tumor cell resistance to ICIs in a HER2-expressing orthotopic breast cancer model. This event was likely related to the improved recruitment of TILs concomitant with enhanced Th1 cell polarization [163]. Recently, D'Amico and colleagues evaluated therapeutic merits and immune-mediated mechanisms of a novel HER2-targeting antibody–drug conjugates (ADCs) bearing a potent anthracycline derivate as payload (T-PNU) in a human HER2-expressing breast cancer model [164]. They found that co-treatment of animals with T-PNU together with anti-PD1 anti-body robustly potentiated tumor regression by increasing CTLs activities [164]. In addition, the study of safety and efficacy of pembrolizumab in combination with trastuzumab and chemotherapy in first-line HER2-expressing metastatic oesophagogastric cancer was conducted between Nov 11, 2016, and Jan 23, 2019, in 37 patients during a phase 2 trial [129]. Achieved results exposed that pembrolizumab could be safely combined with trastuzumab and platinum-based drugs and also had significant activity in HER2-expressing metastatic oesophagogastric cancer [129]. Also, margetuximab, a novel anti-HER2 monoclonal antibody showed acceptable safety, tolerability, and also significant efficacy upon combination therapy with pembrolizumab in 92 patients with HER2-positive gastro-oesophageal adenocarcinoma [165]. Severe treatment-related adverse events were exhibited in 9 of 92 (9%) patients, with no treatment-related deaths. In terms of the efficacy, objective responses were shown in 17 of 92 (18.48%) patients [165]. Ultimately, nivolumab and trastuzumab in combination improved PFS with the manageable safety profile in gastric cancer patients, as reported by Tian et al. [166].

### ICIs with anti-angiogenic agents

**Fig. 3:**
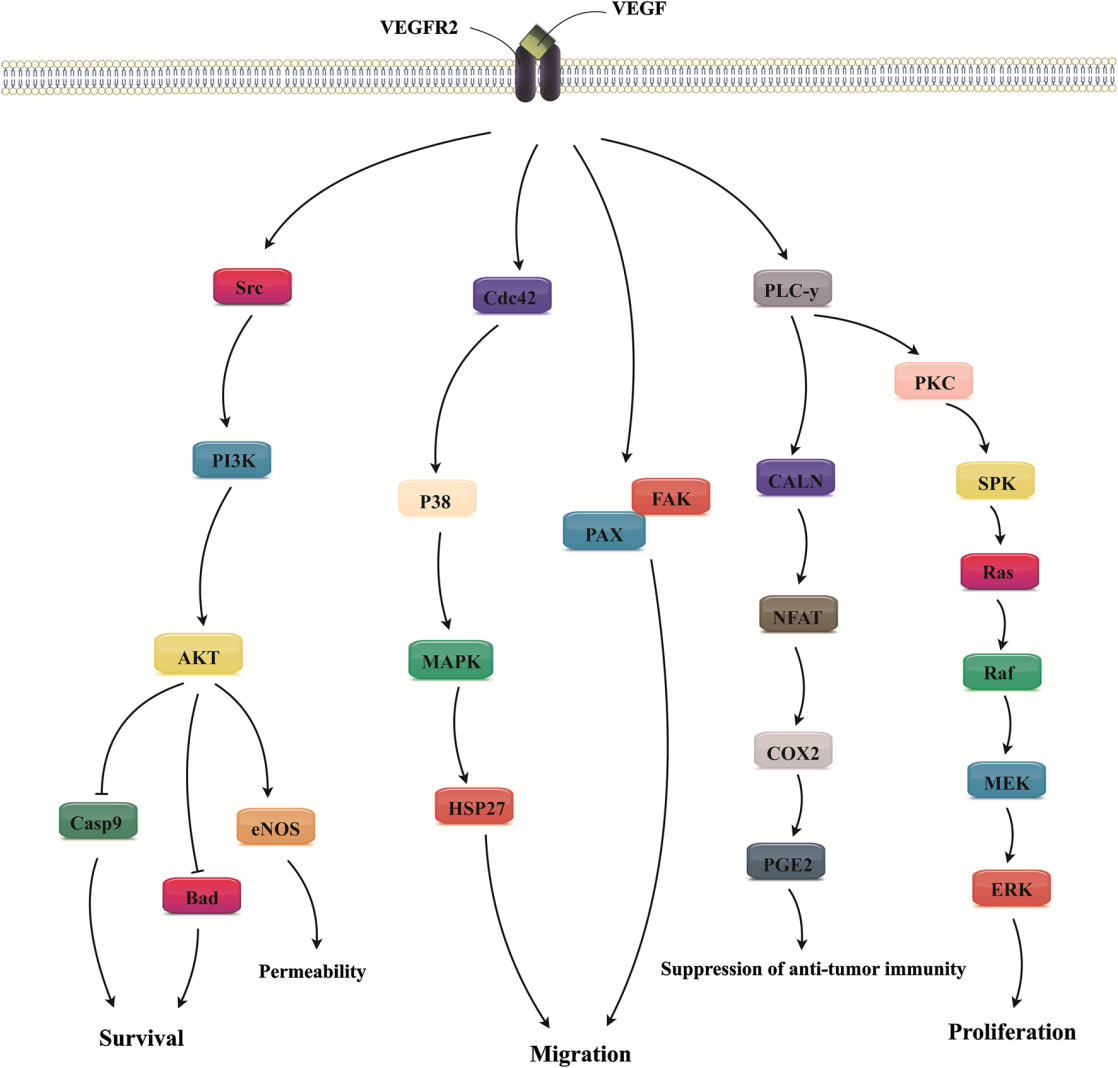
The pivotal role of vascular endothelium growth factor (VEGF) in tumor angiogenesis. The VEGF encourages angiogenesis in tumor cells by interface with responding receptor, VEGFR2, on tumor cells and afterward through activating several signaling axes

Abnormal vasculature is one the most prominent possessions of solid tumors and is complicated in tumor immune escape [167]. This deregulation results from the improvement in the expression of pro-angiogenic factors mainly affecting immune cells both migration and activation [167]. Indeed, anti-angiogenic therapy recently has been developed to fight cancer by abolishing the nutrient and oxygen supply to the tumor cells by a reduction in vascular network and averting the generation of new blood vessels. Given the central role of VEGF signaling in angiogenesis (Fig. 3), the approved angiogenesis inhibitors for tumor therapy chiefly depend on the targeting VEGF actions. Apart from modification of angiogenesis, such drugs can augment immune therapy as a result of the immunomodulatory activities of VEGF [168]. Correspondingly, angiogenesis inhibitors ease alteration of the TME from immunosuppressive to immune-supportive by intensifying the recruitment and induction of immune cells activities. To date, axitinib, bevacizumab, cabozantinib, everolimus, lenalidomide, lenvatinib mesylate, pazopanib, ramucirumab, regorafenib, sorafenib, sunitinib, thalidomide, vandetanib and also Ziv-aflibercept have gained approval from FDA as efficient angiogenesis inhibitors [169]. Bevacizumab as the first FDA-approved VEGF-targeted agent has been indicated for the treatment of a myriad of human tumors, such as CRC, NSCLC, RCC, breast cancer, ovarian cancer, and cervical cancer alone or in combination with other therapeutics [170]. Atezolizumab plus bevacizumab, paclitaxel, and carboplatin have been applied as the first-line treatment of NSCLC patients [171]. Furthermore, bevacizumab plus atezolizumab demonstrated synergistic impact on median OS of RCC patients [172], and also in combination with nivolumab established modest efficacy in ovarian cancer patients [173]. Besides, the safety and also efficacy (improved ORR) of co-administration of PD-L1 inhibitor avelumab with angiogenesis inhibitor axitinib has been evidenced in HCC [174] and also RCC [32] patients during a phase 1b study. Also, co-administration of axitinib plus pembrolizumab caused improved median PFS in patients with sarcoma [168], while combined use of regorafenib plus nivolumab showed a manageable safety profile and also favorable antitumor effects in patients with gastric and CRC [175]. Objective tumor response was detected in 40%, containing gastric cancer (44%) and CRC (36%). Also, median PFS was 5.6 in gastric cancer patients and 7.9 months in patients with and CRC [175]. In contrast, a study in 23 patients with metastatic CRC signified that regorafenib plus nivolumab had no objective response, proposing its non-significant clinical benefits in these patients [176]. Likewise, VEGFR2 inhibitor ramucirumab plus pembrolizumab showed restricted clinical positive effects with infrequent high-grade unwanted effects in patients with advanced BTC [34]. Besides, combined use of ICI and angiogenesis inhibitor lenvatinib supported promoted median OS, but not PFS, than lenvatinib alone in advanced-stage HCC patients [177]. As well, there is clear evidence presenting that addition of the nivolumab to sunitinib or pazopanib could be an effective alternative for the treatment of advanced RCC patients [34, 178].

### ICIs with cancer vaccines (e.g., oncolytic viruses)

Therapeutic cancer vaccines simplify abrogation of tumor progress, eradication of minimal residual disease (MRD), and also inaugurating the durable antitumor memory and ducking untoward reactions [179, 180]. Still, BCG lives, sipuleucel-T (Provenge) and talimogene laherparepvec (T-VEC) are three eminent cancer vaccines authorized by FDA to respectively treat bladder cancer, prostate cancer, and melanoma [181]. T-VEC is the first oncolytic viral immunotherapy, which its direct intratumoral administration stimulates local and systemic immunologic reactions ensuring malignant cell lysis, tracked by secretion of tumor-derived antigens and succeeding induction of tumor-specific effector T-cells [182]. The sipuleucel-T vaccine also was developed respecting the notion of APCs, and thus its administration enables the presentation of tumor-derived antigens in a form that T cells can recognize [183]. Finally, BCG also is a type of immunotherapy vaccine instigating the immune system to fight tumor cells (as shown in bladder cancer) [184].

Various preliminary reports explain that combination therapy with ICIs and cancer vaccines may encourage reinforced immunogenicity and also fence immunosuppressive TME [30]. A recent report has exhibited that co-administration of cancer stem cell (CSC) lysate-pulsed dendritic cell (CSC-DC) with PD-L1 and CTLA-4 inhibitors considerably improved T cell proliferation, inhibited TGF-β secretion, intensified IFN-γ secretion, and finally improved host-specific CD8 + T cell response versus CSCs in B16-F10 mice melanoma tumor model [185]. Similarly, combined use of GM-CSF cell-based vaccines (GVAX) and CTLA-4 inhibitor decreased tumor size and restored the antitumor immune responses in melanoma [29], prostate [186], and also PDA [187] murine model. On the other hand, the DC tumor lysate-based vaccine together with anti-PD-1 anti-body also brought about ameliorated OS in glioma [188] and also lung cancer [189] murine models. In another study, Fu et al. made an IFNγ-inducing cancer vaccine termed TEGVAX that combined GM-CSF and multiple Toll-like receptor (TLR) agonists to raise the frequency of activated DCs [190]. TEGVAX induced tumor regression with stimulated systemic antitumor immunity. Though TEGVAX also surprisingly promoted PD-L1 expression in the TME, the combined use of nivolumab plus TEGVAX provoked complete regression of established tumors [190]. Also, adding the DNA vaccine against murine P815 mastocytoma to CTLA-4 and PD-1 blockade therapy led to the enhanced IFN-γ, IL12, and granzyme B generation in the TME and simultaneously suppressed liver metastasis and improved OS in treated mice [191]. Further, co-administration of TLR5 agonist flagellin-adjuvanted tumor-specific peptide vaccination (FlaB-Vax) with anti-PD-1 mAb inhibited melanoma tumor growth in B16-F10 cell bearing mice [192]. It was found that such desired effects were likely related to the activation of CD8 + T cells and APCs in tumor tissue and also enhanced systemic IFNγ levels [192]. Recently, Yang and coworkers developed a novel vaccine nodule including a simple physical mixture of the peptide nanofibrous hydrogel, PD-1inhibitor, DCs, and tumor antigens [193]. The established vaccine supported a more prominent antitumor effect in tumor models comprising abrogated tumor development and prolonged animal OS as a result of triggering antitumor T-cell immunity [193]. As well, ICIs combination therapy with OVs was found to be able for inducing tumor regression through eliciting anti-tumor M1-like polarization, stimulating recruitment and functions of T effector cells, promoting IFN-γ levels in TME, and ultimately down-regulation of Treg density and activity [194, 195]. Meanwhile, it appears that local viral infection of tumors could circumvent systemic resistance to PD-1-immunotherapy by alteration the diversity of tumor-directed CD8 T-cells in CMT64 lung adenocarcinoma cells bearing murine [196]. Besides, direct measles virus [197] and oHSV expressing IL-12 [198] plus PD-1 and CTLA-4 blockade therapy stimulated tumor regression mainly by inducing the Th1, CTL cells, and M1-macrophages activation in the glioma murine model [194]. Owing to the fact that CD40 agonists make interactions with CD40 molecules on APCs and thereby potentiate their activation to prime tumor-specific CD8 + T cell responses, other studies have focused on CD40L role in inducing antitumor immunity [194]. Correspondingly, co-administration of adenovirus encoding a chimeric, membrane-bound CD40 ligand (ISF35) with PD-1 and CTLA-4 inhibitors caused complete removing of injected tumor cells in the melanoma murine model. Therapeutic effects were accompanied by enhancing the systemic level of tumor-specific CD8 + T cells, and an augmented ratio of intratumoral CTLs to Tregs [194].

Current clinical trials also have signified that DCs-based mRNA vaccination in combination with ipilimumab could stimulate strong CD8 + T-cell responses in stage III or IV melanoma patients [199]. As well, addition of the ipilimumab to GVAX in 30 patients with PDA also resulted in prolonged median overall survival (OS) [200]. In addition, nivolumab plus ISA 101, a synthetic long-peptide human papillomavirus (HPV) vaccine containing HPV-specific T cells, ameliorated median OS and ORR in patients with HPV-16-positive tumors [201].

A summary of conducted studies respecting combination therapy with ICIs and cancer vaccines have been listed in (Tables 2 and 4).

**Table 2:** ICI combination therapy with OVs and other types of cancer vaccines

Tumor	Target IC	Agent (s)	Result (s)	References
Glioma	CTLA-4 PD-1	IL-12-oHSV	Induction M1macrophage and T effector (CD4 + and CD8 + T cells) function along with suppression of Treg	[198, 254]
Melanoma	CTLA-4 PD-1	PLG	Promotion of CTL activity and inducing tumor regression	[255]
Rectal cancer Osteosarcoma	PD-1	hTERT-oAd	Hindrance of tumor regression by recruitment of CTLs	[256]
Breast cancer	PD-1 CTLA-4	sTGFβRIIFc-oAd	Abrogation of tumor development and lung and liver metastases	[257]
HER-2 positive tumors	PD-1 PD-L1	HER-2 B-cell peptide vaccine	Robust abrogation in tumor growth	[31]
Melanoma	PD-L1 CTLA-4	CSC-DC	Enhancing T cell proliferation, suppressing TGF-β secretion, promoting IFN-γ secretion, and finally triggering specific CD8 + T cell response against CSCs	[185]
Lung cancer Breast cancer Melanoma Lymphoma	PD-1 PD-L1 CTLA-4	GM-CSF-oHSV	Tumor regression and also stimulation of immunological memory	[257]
Melanoma	PD-1	T-VEC	Hindrance of tumor growth by enhancing the infiltration of CTLs, reducing intratumoral Tregs, and activation of Th1 in the TME	[258]
Melanoma	CTLA-4 PD-1	Ovalbumin	Delay in tumor growth and extended OS rate of mice by increased intratumoral CD8 + infiltration	[259]
Glioma	PD-1	ZIKV	Better OS rate of treated mice	[260]
Rhabdomyosarcoma	PD-1	oHSV	Improving T effector (CD4 + and CD8 + T cells) function along with suppression of Treg	[261]
Melanoma	PD-L1	oHSV	Improving IFNγ-producing CD8 + TILs activities, and promoted OS rate	[262]
Melanoma	PD-1	Archaeosome-OVA	Robust tumor recession	[263]
Glioma	PD-1	EGFR- MV	Recruitment and infiltration of TILs into the brains of treated mice, and also improved OS rate	[197]
Lung cancer	PD-1	oAd	Reserve of tumor cell development mediated by activation of CTL	[196]
Lung cancer	PD-L1	Lm-LLO-E6	Stimulation of prolonged OS rate	[264]
Melanoma	PD-1 PD-L1 CTLA-4	CD40L- oAd	Boosting the systemic level of tumor-specific CD8 + T cells, and also augmentation of the ratio of intratumoral CD8 + T cells to Treg	[194]
Glioma	PD-L1	CD40L- oAd	Reserve of tumor growth accompanied with increased OS rate	[265]
Prostate cancer	PD-1	oAd	Stimulation of antigen-specific CD8 + T-cell responses	[266]
Oral cancer	CTLA-4	HPV E6/E7 peptide	Promoted intratumoral levels of CD8 T cells concomitant with reduced MDSCs and Treg	[267]
Melanoma	PD-1	Reovirus	Activation of and CTL along with abridged Treg activity	[268]
Glioma	PD-1	Reovirus	Promoting the expression of IFN-regulated gene expression	[269]
Melanoma	PD-1	oAd	Abrogated tumor growth accompanied with improved OS rate	[270]
Melanoma	PD-1	FlaB-Vax	Significant rise in tumor-infiltrating effector memory CD8 + T cells and systemic IFNγ levels	[192]
Melanoma	PD-1 CTLA-4	Ovalbumin	Induction of CD8 + T cells activities associated with enhanced eliminated tumor cells	[271]
Melanoma	PD-L1	MV	Stimulation of tumor regression	[272]
Prostate cancer	PD-1	VLP	Reduced tumor burden by activating CTLs	[273]

*ICI* immune checkpoint inhibitor, *PD-1* programmed cell death protein 1, *PD-L1* programmed death-ligand 1, *CTLA-4* cytotoxic-T-lymphocyte-associated protein 4, *CTLs* cytotoxic T cells, *IFN* interferon, *Tregs* regulatory T cells, *TME* tumor microenvironment, *TILs* tumor-infiltrating lymphocytes, *APC* antigen-presenting cell, *MDSC* myeloid-derived suppressor cells, *OS* overall survival, *TGF-β* transforming growth factor, *DC* dendritic cell, *CSC* cancer stem cell, *oHSV* oncolytic herpes simplex virus, *oAd* oncolytic adenovirus, *MV* measles virus, *VSV* Vesicular Stomatitis Virus, *ZIKV* Zika virus, *FlaB-Vax* Flagellin-adjuvanted tumor-specific peptide vaccination, *HER2* human epidermal growth factor receptor 2, *VLP* virus-like particles, *HPV* human papillomavirus, *EGFR* epidermal growth factor receptor, *hTERT* human telomerase reverse transcriptase, *TGFβRIIFc* transforming growth factor-beta receptor 2 fused with Fc protein, *GM-CSF* Granulocyte–macrophage colony-stimulating factor

### ICIs with radiation therapy (RT)

Radiotherapy (RT) is employed generally as a standard treatment for more than 50% of patients suffering from tumors [202]. The abscopal influences elicited by local RT, which is defined as systemic anti-tumor immune reactions, enable the removing non-irradiated metastatic lesions at a distance farther from the primary area of irradiation [203]. As the ICIs can improve the systemic anti-tumor reactions of RT, combined use of RT and immunotherapy has recently attracted widespread attention [1]. The stimulation of immunogenic cancer cell death is the common mechanism for most RT plans. Then, the DCs are stimulated by the secreted danger signals and by taking up tumor peptides established by irradiated cells, and in turn, facilitates DCs-dependent T cells activation [1].

Studies have reported that RT in combination with targeting CTLA-4 and/or PD-1/PD-L1 could provoke CTLs-mediated anti-tumor immunity [28]. For instance, in glioma xenograft-bearing mice, combination therapy with PD-1 blockade and dose brain-directed radiation (10 Gy) resulted in anti-tumor impacts with a 75% complete pathologic response and also substantially improved OS mainly caused by activation of CTLs and macrophages [204]. Meanwhile, RT seemed to stimulate macrophage repolarization, enhancing M1/M2 ratio [204]. However, other reports revealed that RT combined with anti-PD-1 treatment might lead to more severe lung injury in the tumor cell-bearing mice, attended by boosted neutrophil infiltration and enhanced inflammatory response [205]. Thereby, tight consideration must be taken during this combination therapy to ameliorate the safety profile. Further, LM8 osteosarcoma cells bearing mice irradiated with either carbon ions or x-rays along with PD-1 and CTLA-4 inhibitors experienced abrogated growth of the abscopal tumors, which was mediated by increased CD8 + cells unlike mice treated with RT or ICI alone [206]. The achieved results indicated that adding high-energy carbon ion radiation therapy to ICI can be considered as an efficient plan for the treatment of advanced tumors [206]. RT (20 Gy) plus PD-1 or PD-L1 blocked therapy also robustly potentiated OS rate in castration-resistant prostate cancer (CRPC) preclinical model than monotherapy with each agent [207]. Meanwhile, the median OS for anti-PD-L1 monotherapy was 13 days versus 30 days for anti-PD-L1 plus RT, and anti-PD-1 monotherapy was 21 days versus 36 days for anti-PD-1 plus RT [207].

In this regard, a trial conducted, between February 2016 and December 2017, on 124 patients with advanced NSCLC verified the safety and efficacy of combination therapy with nivolumab and RT [208]. Results revealed that previous RT could be an independent prognostic marker of promising prognosis after nivolumab therapy and also could improve the ORR to nivolumab treatment [208]. In this trial, ORR was enhanced from 19% (RT group) and 28% (nivolumab group) to 36.4% (RT plus nivolumab group) [208]. Likewise, patients (e.g., NSCLC and HNSCC) treated with PD-1/PD-L1 blocked therapy could benefit from local RT, as evidenced by longer PFS and OS [209]. Similarly, the combination of ICI and RT supported enhanced OS, PFS, and disease control rate (DCR) in patients with NSCLC and lung cancer [210]. The addition of the hypofractionated body radiotherapy (H-RT) to nivolumab or ipilimumab also was found that be safe and also served therapeutic merits in melanoma and RCC patients [211]. Other trials also have verified the safety of RT plus ICI in tumor patients with manageable ir-AEs [212, 213]. Nonetheless, Pike et al. have found that extracranial or prolonged regimen of RT might augment the risk of severe lymphopenia, accompanied by poorer survival in patients treated with ICI [214].

### ICIs with ACT

Adoptive cell therapy (ACT) with using TILs or gene-modified T cells expressing novel T cell receptors (TCR) or chimeric antigen receptors (CAR) is another tactic to inspire the immune system to induce and so detect maligned cells and eradicate them [215, 216]. Responders to ICI therapy usually suffer from T cell-inflamed tumors, reflecting the significance of evolving approaches that adapt non-T cell-inflamed tumors to T cell-inflamed tumors. There is some report indicating that co-administration of anti-PD-L1 antibody plus TILs might enhance T cell infiltration and IFN-γ production in tumor cell-bearing mice, underlying delayed tumor growth [217]. As well, a clinical trial in 13 patients with metastatic melanoma revealed that combined use of ipilimumab (3 mg/kg) plus TIL might induce significant ORR (38.5%) and promoted PFS (7.3 months) [218]. Co-administration of the TILs with ipilimumab or nivolumab into 6 patients with ovarian cancer also supported a partial response in 1 patient, while 5 others experienced disease stabilization for up to 1 year [219]. In another report, targeted delivery of PD-1-blocking single-chain variable fragments (scFv) by CAR-T cells potentiated anti-tumor immunity in vivo, as evidenced by Rafiq et al. reports [220].

In 2017, Shaw et al. found that in HNSCC murine models, co-administration of HER2-redirected CAR-T cell plus PD-1 inhibitor substantially improved survival compared to monotherapy with each of them [221]. Similarly, anti-EGFR variant III CAR-T cell therapy in association with anti-PD-1 mAb could exert more efficient and persistent therapeutic influences on GBM and also stimulate an intensified number of TILs in vivo [222]. Besides, combined use of the mesothelin-specific CAR-T cells with PD-1 inhibitor exhibited substantial safety and modest efficacy (as shown by improved OS) in 18 patients with MPM [223, 224]. However, it seems that the execution of large-scale studies is required to address the reliable efficacy of this intervention in MPM patients. Besides, anti-CD19 CAR T cells plus pembrolizumab enhanced and/or prolonged detection of circulating CAR T cells and also resulted in ORR (50%) in leukemia patients (NCT02374333, NCT02906371) [225].

### ICIs with CXCR4 inhibitors

Overexpression of C-X-C chemokine receptor (CXCR) 4 is allied with undesired prognosis in human several tumors [226, 227]. Hence, CXCL12 (SDF-1)/CXCR-4 signaling pathway has been described as a rational and effective therapeutic target in the context of tumor therapy due to its pivotal role in tumor instigation and development by triggering various signaling pathways, comprising ERK1/2, Ras, JNK and p38 MAPK along with adjusting CSCs [228]. As a result, CXCL12/CXCR4 antagonists have currently been developed to impair pathological procedures and also disrupt cancer cell adhesion to the stromal cells [229, 230]. Disrupting such adhesions ultimately facilitate the cancer cells' mobilization into the systemic circulation and can offer an appreciated opportunity to eradicate these cell by other modalities, such as cytotoxic chemotherapeutic agents [231]. Recent reports displayed that promotion of the CXCL12 expression in HCC models improved hypoxia, and also induced the recruitment of immunosuppressive cells, whereas PD-1 inhibitor along with CXCR4 inhibition and sorafenib reduced HCC growth [231]. Dual targeting CXCR4 and PD-1 also sustained the TILs population as well as their activation in the glioma microenvironment [36]. Targeting MDSCs with CXCR4 blockade potentiated anti-PD-1 to uphold antitumor immune reactions and ameliorated OS in glioma cell-bearing mice [36]. Another important study has demonstrated that tumor-infiltrating MDSCs usually are CXCR4 positive and could migrate toward the CXCL12 gradient [232]. Given that CXCL12/CXCR4 interaction leads typically to the induction of the AKT pathway and afterward compromises MDSCs apoptosis, Jiang and coworkers suggested that plerixafor (AMD3100), a highly specific CXCR4 antagonist, could provoke a synergistic influence with anti-PD-1 antibody to enable tumor regression in a murine model of osteosarcoma [232]. Also, addition of AMD3100 to PD-1 inhibitor potently delayed tumor development and prolonged OS in ovarian cancer murine model more prominently than single-agent administration [233]. Furthermore, the intervention was accompanied by augmented effector T-cell infiltration as well as function concomitant with heightened memory T cells in TME [233]. Combination therapy also resulted in reduced intratumoral Tregs and also MDSCs allied with reduced IL-10 and IL-6 in the ascites and simultaneously induced M2-to-M1 macrophage polarization in the tumor [233]. Of course, some reports signified that CXCR4 blockade might stimulate the proportion of circulating myeloid cells during active treatment in the ovarian cancer mice model, thereby additional examination into this novel therapeutic method is warranted [234]. On the other hand, dual-targeting PD-L1 and CXCR4 showed an amplified antitumor outcome, reduced Tregs infiltration, and extended OS compared with monotherapies in 4T1 TNBC [235], MC38 colon cancer [236] and B16 melanoma cell [236] xenografts. These data offered proof of the concept that CXCR4 inhibitors have pronounced capacities to expand ICI therapies to originally ICI-insensitive tumor types. Further, treatment of fresh human PDAC specimens with PD-1 and CXCR4 inhibitors gave rise to enhanced tumor cell death and also lymphocyte expansion [237]. Also, another clinical trial (NCT04543071) is ongoing to address the safety and efficacy of combination therapy with chemotherapeutic agents (gemcitabine and nab-paclitaxel) with CXCR4 inhibitor (motixafortide), and PD-1 inhibitor (cemiplimab) in patients with metastatic PDAC [238]. Besides, combined use of mavorixafor (X4P-001) as an allosteric CXCR4 inhibitor plus nivolumab in 9 patients with advanced RCC showed acceptable antitumor effect and a manageable safety profile (NCT02923531) [239, 240]. As well, it was supposed that enhancement in levels of CXCL9 correlates with clinical benefit [239].

A summary of conducted studies respecting combination therapy with ICIs and CXCR4 blockade therapy have been listed in (Tables 3 and 4).

**Table 3:** ICI combination therapy with CXCR4 blockade in cancer therapy

Tumor	Target IC	Result	References
Hepatocellular carcinoma	PD-1	Inhibition of tumor growth and lung metastasis along with improved OS rate in mice models	[231]
Triple-negative breast cancer	PD-L1	Robust antitumor effect and extended OS rate in 4T1 cell bearing murine model	[235]
Ovarian cancer	PD-1	Enhancing the effector T-cell infiltration, improving effector T-cell function and also memory T cells in TME Reducing intratumoral Treg cells and promoting the conversion of Treg cells into T helper Improved OS rate in mice model	[233]
Glioblastoma	PD-1	Improving the memory T cells and reducing MDSCs Promoting CD4 + /CD8 + ratios in the brain and elevation of pro-inflammatory cytokines levels in the brain	[36]
Pancreatic ductal adenocarcinoma	PD-1	Inspiring the CD8 + T-cell migration into the juxtatumoral compartment and also induction apoptosis in tumor cell	[237]
Osteosarcoma	PD-1	Inducing tumor regression by suppressing MDSCs in mice model	[232]
Colon cancer Melanoma	PD-1	Inhibition of tumor growth in two syngeneic murine models, by improving granzyme and suppressing FOXP3 cells infiltration	[236]
Ovarian cancer	PD-1	Improved OS rate in treated mice model	[234]
Lung cancer	PD-L1	Improving the T cell infiltration, enhancing expression of calreticulin on tumor cells Reducing MDSCs and Treg in the TME	[274]
Glioblastoma	PD-1	Demonstrating immune memory concurrently reducing populations of MDSCs and tumor-promoting immune cells Improved OS rate in treated mice model	[275]
Triple-negative breast cancer	PD-L1	Promoting the tumor immunogenicity to recruit T cells, attenuating the physiological barricades of intratumoral fibrosis and collagen to support T cell infiltration, and reducing the immunosuppressive cells to revive T cells	[276]
Melanoma	PD-1	Modulating the immune cell profile within the TME and improving CD8 + T cell infiltration	[277]

*ICI* immune checkpoint inhibitor, *PD-1* programmed cell death protein 1, *PD-L1* programmed death-ligand 1, *CTLs* cytotoxic T cells, *IFN* interferon, *Tregs* regulatory T cells, *TME* tumor microenvironment, *MDSC* myeloid-derived suppressor cells, *OS* overall survival, *FOXP3* Forkhead box protein P3

**Table 4:** Clinical trials result based on combination therapy with ICIs and other modalities

Tumor	Agent (s)	Result (s)	References
ICI plus Anti-anti-angiogenic agent			
Triple-negative breast cancer	SHR-1210 plus Apatinib	Notable tolerability and efficacy Higher TGF-β expressions associated with favorable prognosis	[278]
Renal cell carcinoma	Atezolizumab plus Bevacizumab	Enhancement in intratumoral CTL cells, and also intra-tumoral MHC-I, Th1, and T-effector markers, and CX3CL1	[279]
Melanoma	Ipilimumab plus Bevacizumab	Remarkable safety and tolerability Modification in tumor vasculature and immune responses and alteration of lymphocyte trafficking, and immune regulation	[280]
Ovarian cancer	Nivolumab plus Bevacizumab	Anti-tumor activity, in particular, in the platinum-sensitive setting	[173]
Renal cell carcinoma	Nivolumab plus Sunitinib	Remarkable irAEs along with no improvement in the OS	[281]
Colorectal cancer	Atezolizumab plus Bevacizumab	Without unexpected adverse events or severe toxicities	[282]
Renal cell carcinoma	Pembrolizumab plus Axitinib	Notable tolerability and efficacy along with no unexpected toxicities	[33]
Melanoma	Ipilimumab plus Bevacizumab	Improved OS	[283]
Sarcoma	Nivolumab plus Sunitinib	Improved PFS	[284]
Non-small cell lung carcinoma	Sintilimab plus Anlotinib	Robust efficacy, durability, and safety profile Improved PFS	[285]
Advanced solid tumors	Pembrolizumab plus Lenvatinib	Manageable safety profile and favorable antitumor activity	[286]
Renal cell carcinoma	Nivolumab plus Cabozantinib	Improved PFS and OS	[287]
Lymphoma Solid tumors	Ipilimumab and Lenalidomide	Significant tolerability concomitantly preliminary signals of anti-tumor activity	[288]
Non-small cell lung carcinoma	Nivolumab plus Bevacizumab	Improved PFS and ORR	[289]
ICI plus Chemotherapeutic agent			
Non-small cell lung carcinoma	Nivolumab plus Ipilimumab and Platinum-based compound	Improved OS versus chemotherapy alone and also favorable risk–benefit profile	[290]
Solid tumors	Cemiplimab plus RT and CTX	Acceptable safety but no efficacy	[268]
Non-small cell lung carcinoma	Pembrolizumab plus Carboplatin and Pemetrexed	Improved OS and PFS	[291]
Non-small cell lung carcinoma	Nivolumab plus Platinum-based compound	Improved OS	[291]
Non-small cell lung carcinoma	Ipilimumab plus Paclitaxel and Carboplatin	Improved OS and PFS with manageable irAEs	[292]
Mesothelioma	Nivolumab plus Cisplatin and Pemetrexed	Some irAEs such as severe abdominal distention	[293]
Pancreatic cancer	Ipilimumab plus Gemcitabine	No superiority over chemotherapy with gemcitabine	[294]
Biliary tract cancer	Nivolumab plus Gemcitabine and Cisplatin	Improved OS and PFS with manageable irAEs FasL, MCP-1, and INF-γ associated with favorable prognosis	[149]
Pancreatic ductal adenocarcinoma	Nivolumab (Nivo) plus nab-Paclitaxel and Gemcitabine	Improved OS along with severe irAEs such as pneumonitis in some case	[295]
Urothelial cancer	Pembrolizumab plus Docetaxel or Gemcitabine	Improved PFS and ORR	[296]
Melanoma	Ipilimumab plus Dacarbazine	No tolerability along with high-grade liver toxicities	[297]
ICI plus Radiotherapy			
Melanoma	Ipilimumab plus RT	Synergetic anti-tumor response	[298]
Melanoma	Ipilimumab plus RT	A systemic complete response	[299]
Prostate cancer	Ipilimumab plus RT	Complete response in 1 participant only	[300]
Advanced solid tumors	Nivolumab plus Ipilimumab and RT	Acceptable tolerability along with manageable irAEs	[212]
Advanced solid tumors	Durvalumab plus RT	Acceptable tolerability without abscopal effect	[301]
Renal cell carcinoma Melanoma	Nivolumab plus Ipilimumab and RT	Significant improvement in ORR and OS Any grade irAEs in 46 of 59 patients	[211]
Non-small cell lung carcinoma	Pembrolizumab plus RT	Improvement in ORR and OS with an acceptable safety profile	[91]
ICI plus Cancer vaccines			
Melanoma	Ipilimumab plus T-VEC	Improved ORR	[302]
Melanoma	Ipilimumab plus T-VEC	Improved ORR	[303]
Prostate cancer	Ipilimumab plus Sipuleucel-T	Acceptable tolerability	[304]
Prostate cancer	Ipilimumab plus Sipuleucel-T	Improved OS	[304]
Prostate cancer	Ipilimumab plus GVAX	Improved OS	[305]
Prostate cancer	Ipilimumab plus GVAX	Manageable irAEs	[306]
Pancreatic ductal adenocarcinoma	Ipilimumab plus GVAX	Prolonged disease stabilization and a trend of favorable median OS	[200]
Melanoma	Ipilimumab plus Peptide vaccine	Durable ORR	[307]
Melanoma	Ipilimumab plus Peptide vaccine	No difference in median OS	[308]
Melanoma	Pembrolizumab plus T-VEC and RT	No significant effect	[309]
Melanoma	Nivolumab or Ipilimumab plus T-VEC	Potentiating the antitumor effect of T-VEC	[310]
Pancreatic ductal adenocarcinoma	Nivolumab plus GVAX and CTX	Improved ORR without any effect on OS	[311, 312]
Melanoma	Nivolumab plus Gp100	Acceptable tolerability	[313]
ICI plus Other modalities			
Triple-negative breast cancer	Durvalumab plus Olaparib	Acceptable tolerability along with preliminary activity in recurrent cancers	[314]
Ovarian cancer	Durvalumab plus Olaparib	Modest clinical activity	[315]
Melanoma	Pembrolizumab plus Dabrafenib and Trametinib	Enhanced anti-tumor responses	[316]
Renal cell carcinoma	Nivolumab plus Mavorixafor	Potential antitumor activity and a manageable safety profile	[239]

*ICI* immune checkpoint inhibitor, *CTLs* cytotoxic T cells, *IFN* interferon, *OS* overall survival, *ORR* objective response rate, *PFS* progression-free survival, *irAEs* immune related adverse events, *MCP-1/CCL2* monocyte chemoattractant protein-1, *RT* radiotherapy

## Conclusion and prospect

As shown in clinical trials (Tables 4 and 5), addition of ICIs to other therapeutic means has been shown encouraging outcomes to treat even metastatic tumors with unfavorable prognosis. However, the intervention-associated irAEs can hurdle their application in the clinic. Skin and colon are the most common organs, while the normal activity of lungs, kidneys, liver, and also heart mainly impaired by ICIs alone or in combination therapies [241]. Though, corticosteroids are usually exploited to ameliorate moderate and severe irAEs, additional immunosuppressive drugs may sometimes be prerequisite [242, 243]. Also, much efforts have recently been spent to determine predictive biomarkers for ICIs response [244]. Meanwhile, PD-L1 expression, microsatellite instability (MSI), high tumor mutational burden (TMB) along with CD8 infiltrates are noted as foremost predictive markers for ICIs response [245–247]. Taken together, we propose that fulfilling of large-scale trials with further attention to the predictive biomarkers can durably arouse more preferred outcomes with manageable irAEs.

**Table 5:** A summary of clinical trials based on combination therapy with ICIs plus other modalities in human cancers registered in https://clinicaltrials.gov (October 2021)

Condition	Agents		Study phase	Participant number	Study location	NCT number
Non-small-cell lung carcinoma	Platinum + Durvalumab	2		55	USA	NCT04062708
Solid tumor Hematological malignancy	Eliglustat + ICI	1		30	China	NCT04944888
Advanced tumors	Ipilimumab, Nivolumab, Pembrolizumab + BBI608	1/2		104	USA	NCT02467361
Non-small-cell lung carcinoma	Tocilizumab + Atezolizumab	1/2		28	USA	NCT04691817
Non-small-cell lung carcinoma	Platinum + angiogenesis inhibitors and ICI	NA		126	China	NCT04137588
Hepatocellular carcinoma Biliary tract cancer	Nivolumab + Pembrolizumab	NA		100	Republic of Korea	NCT03695952
Pancreatic cancer	RT + ICI	1/2		52	USA	NCT04327986
Advanced solid tumors	ASP8374 + Pembrolizumab	1		169	USA	NCT03260322
Solid tumor Lymphoma	Ad-p53 Gene Therapy + ICI	2		40	USA	NCT03544723
Multiple primary lung cancer	Microwave ablation + Camrelizumab	2		146	China	NCT05053802
Advanced solid tumors	FT500 + ICI	1		76	USA	NCT03841110
Advanced solid tumors	DSP-7888 Dosing Emulsion + ICI	1/2		84	USA	NCT03311334
Intrahepatic cholangiocarcinoma	ICI + Lenvatinib and Sintilimab	2		25	China	NCT05010681
Solid tumors	Gut Microbiome + ICI	NA		800	USA	NCT05037825
Non-small-cell lung carcinoma	ICI + OSE2101, Docetaxel, Pemetrexed	3		363	USA	NCT02654587
Genitourinary cancer Melanoma	Infliximab or Vedolizumab + ICI	1/2		100	USA	NCT04407247
Non-small-cell lung carcinoma	Pembrolizumab + RT	1/2		164	International	NCT03996473
Non-small-cell lung carcinoma	Ramucirumab + Atezolizumab	2		21	USA	NCT05007769
Non-small-cell lung carcinoma	Ipilimumab + Nivolumab	3		1360	France	NCT03469960
Renal cell carcinoma	Atezolizumab + Cabozantinib	3		500	International	NCT04338269
Cervical cancer	BAVC-C + Durvalumab	2		37	Republic of Korea	NCT04800978
Cervical cancer	Pembrolizumab + Platinum and RT	1		1	United Kingdom	NCT03144466
Squamous cell carcinoma of head and neck	Nivolumab + Surgical resection	2		24	USA	NCT03878979
Non-small-cell lung carcinoma	Atezolizumab + RT	1		2	USA	NCT02599454
Advanced solid tumors	Nivolumab + Copanlisib	1/2		102	USA	NCT04317105
Inoperable esophageal Cancer	Nivolumab, Ipilimumab + Chemoradiation	2		103	France	NCT03437200
Non-small-cell lung carcinoma	Ramucirumab + SAR408701	2		36	USA	NCT04394624
Hepatocellular carcinoma	Pembrolizumab + Regorafenib	2		119	USA	NCT04696055
Lung cancer	Pembrolizumab + Idelalisib	1/2		40	USA	NCT03257722
Metastatic colorectal cancer	Atezolizumab + Bevacizumab and RT	2		52	France	NCT04659382
Advanced solid cancers	Ipilimumab, Nivolumab + Copanlisib Hydrochloride	1/2		102	USA	NCT04317105
Esophageal cancer	Nivolumab, Ipilimumab + Chemoradiation	2		130	France	NCT03437200
Non-small-cell lung carcinoma	Ramucirumab + Atezolizumab	2		21	USA	NCT03689855
Castration-resistant prostate cancer	Pembrolizumab + HER2Bi-armed	2		33	USA	NCT03406858
Advanced solid tumors	ICI + RT	NA		200	Germany	NCT04892849
Liver-dominant Metastatic colorectal cancer	Atezolizumab + RT, Bevacizumab	2		52	France	NCT04659382

*ICI* immune checkpoint inhibitor, *RT* radiotherapy

## Data Availability

Not applicable.
